# Factors affecting diagnosis and management of hypertension in Mazowe District of Mashonaland Central Province in Zimbabwe: 2012

**DOI:** 10.1186/1471-2261-14-102

**Published:** 2014-08-19

**Authors:** More Mungati, Portia Manangazira, Lucia Takundwa, Notion T Gombe, Simbarashe Rusakaniko, Mufuta Tshimanga

**Affiliations:** 1 Department of Community Medicine, University of Zimbabwe, Mount Pleasant, Harare, Zimbabwe; 2 Ministry of Health and Child Care, Harare, Zimbabwe

## Abstract

**Background:**

From 2005 to 2011 Mazowe District recorded a gradual decline in prevalence of hypertension in the face of rising incidence of complications like stroke. This raised questions on whether diagnosis and management of hypertensive patients is being done properly.

**Methods:**

We conducted an analytic cross sectional study at three hospitals in Mazowe District where we randomly selected 201 of 222 patients from out patients departments and interviewed a convenience sample of 23 healthcare workers. Structured interviewer administered questionnaires were used to collect data on demographic characteristics and knowledge from patients, as well as knowledge and practices from health workers. Physical measurements were done on all patients. Frequencies; proportions, odds ratios, Chi square test and stratified & logistic regression analysis were done using Epi info version 3.5.4 while graphs were generated using Microsoft excel®. Calculations were done at 95% confidence interval.

**Results:**

Prevalence, awareness, control, compliance, and complication rate of hypertension were: 69.7%, 56.2%, 22.0%, 59.8% and 20.7% respectively. Independent risk factors for hypertension were age (POR 3.09; 95% CI: 1.27-7.5), obesity (POR 4.37; 95% CI: 1.83-10.4), and previous high blood pressure reading (POR 19.86; 95% CI: 8.61-45.8). Complications included cardiac failure (8.6%), visual defects (4.3%) and stroke (3.6%). Co-morbid human immunodeficiency virus (10.7%) and diabetes mellitus (12.1%) were identified among respondents. Knowledge was poor in 47.7% of health workers.

**Conclusions:**

Risk factors found in this study are consistent with other studies. Health service factors are the main reasons for poor diagnosis and management of hypertension. Health workers need training on diagnosis and management of hypertension. Guidelines, digital sphygmomanometers and adequate drug supply are needed. District has since purchased digital BP machines and requested assistance with training on clinical features of hypertension, use of digital machines, and how to properly measure BP. A policy document on non-communicable diseases including hypertension was subsequently developed by the Ministry of Health and Child Care and currently awaiting endorsement by parliament.

## Background

Hypertension is a chronic, sometimes acute, condition characterized by an abnormally raised blood pressure resulting in end organ damage. Hypertension is strictly limited to arterial blood pressure. Normally the maximum pressure exerted via blood on the arterial walls by the heart during a contraction (systolic pressure), is below 140 mmHg while the minimum pressure on the arterial wall when the heart is relaxing between contractions is below 90 mmHg (diastolic pressure).

When the cause is unknown it is designated essential hypertension. This has a genetic link within families. Secondary hypertension is where the cause is identified as in: chronic kidney disease; adrenal gland disorders; pregnancy; or drug induced hypertension [1].

Zimbabwe is experiencing an epidemiological transition where the prevalence of hypertension is rising due to Western diets and lifestyle [2].

The impact of hypertension was previously ignored as institutions focused on communicable diseases (NCDs) such as HIV. With these communicable diseases becoming more controlled and life expectancy increasing, importance of NCDs is now being felt [3]. Because it is mainly asymptomatic people can develop complications without knowing the cause is hypertension. Those who develop symptoms usually complain of any of: headaches, visual disturbances, nausea, vomiting and epistaxis or confusion [4].

Risk factors for developing hypertension can be modifiable (as in obesity, stress, excess dietary salt, physical inactivity), or non-modifiable (increasing age, black race, family history, and female sex).

Hypertension tends to rise with increasing age due to the stiffening of the arterial walls [5]. Females usually have a higher body mass index (BMI) which is associated with higher prevalence of hypertension [6,7]. In addition, higher social classes and living in urban areas have been identified as risk factors elsewhere [8].

Zimbabweans have adopted unhealthy eating habits and behaviours such as smoking cigarettes and alcohol abuse. According to the STEPwise survey done in 2005, 58% of men and 13.5% of women took alcohol while 33.4% of men and 5% of women used tobacco [9]. The result has been a rise in case fatality rate due to hypertension from 2.8% in 1990 to 8% in 1997 [10].

Hypertension is diagnosed by measuring BP using a sphygmomanometer. In Zimbabwe, three consecutive readings at least 4 hours apart are used for diagnosis after which a Medical Doctor can commence treatment. However a single systolic blood pressure (SBP) above 180 mmHg or a diastolic blood pressure (DBP) above 110 mmHg is indication for treatment [11].

**Figure 1:**
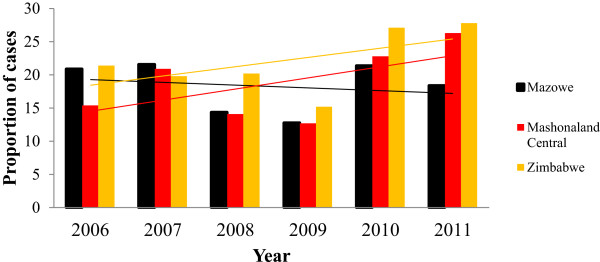
Trend in the prevalence of hypertension in Mazowe District, Mashonaland Central Province and Zimbabwe: 2006 – 2011.

Trend analysis of hypertension between Mazowe District, Mashonaland Central Province and Zimbabwe from 2004 to 2011 showed that the district trend is slowly declining out of keeping with the national trend (Figure 1). Data from the T5 submitted through the District Health Information Software (DHIS) showed that the prevalence of hypertension in Mazowe district in 2011 was 15% which is lower than that of Zimbabwe standing at 27%.

Cases of stroke have been rising at Concession hospital from 2005 to 2011. Anecdotal evidence suggested that management of hypertensive patients is poor and patients may not be taking their medication as prescribed. So we decided to investigate the reasons for the low prevalence of hypertension in Mazowe District and to find out if patients were being managed properly once diagnosis has been made.

## Methods

An analytic cross sectional study was conducted among randomly selected out-patients attendees at three hospitals (Concession, Mvurwi and Howard). Those aged 18 years and older, residing in Mazowe District, consented and were not pregnant were selected using the lottery method with the OPD registration book being the sampling frame. After registration of patients there was a lag period before consultation. During this period we noted the range of OPD numbers in the registration book sequenced from number one until the last numbered patient. We then used previously prepared numbered card for selection. The cards were put in a hat and raised above the level of the head. The card would then be picked up without replacement until we reached the sample size for that hospital. Those patients who agreed to be interviewed would then be enrolled into the study. Using the Dobson formula we calculated a sample size of 196 at 95% confidence interval, with a precision of 0.05 assuming 15% of respondents will be hypertensive (the proportion of patients diagnosed with hypertension in Mazowe District in 2011 as reported through the DHIS).

We also interviewed a convenience sample of 23 health care workers using a structured questionnaire. The questionnaire focused on demographic details of health workers, their knowledge on hypertension and their practices when faced with a patient who has hypertension. Key informants interviewed were the District Health Executive members.

A structured interviewer administered questionnaire was used to collect information from patients followed by direct measurements of: BP, weight, height, waist and hip circumference, and random blood sugar (RBS). The questionnaire collected information on socio-demographic, socio-economic and socio-cultural information. Information on patient knowledge of hypertension and their perception on health services available were also sought. Further information of risk factors (modifiable and non-modifiable), how hypertension was being managed by health care workers and patient compliance to medication was looked for during interviews.

BP was measured using a digital BP monitor *(CITIZEN*™ *Blood Pressure Monitor CH-411C – Japan CBM Corporation)* and classified using the British Hypertension Society guidelines as Zimbabwe has no published guidelines for classifying Hypertension. Each patient rested for at least 5 minutes prior to BP measurements during interview while sitting in a chair with both feet flat on the floor. Both arms were supported at the level of the heart on a table. BP was then measured at the end of the interview. Readings were taken from both arms then another reading taken from the side with the higher reading. There was a 2 minute lag period between readings. The average of the two readings from the same arm was recorded and classified as shown in Table 1.

**Table 1:** Classifications of hypertension

**Classification**	**Systolic blood pressure (mmHg)**	**Diastolic blood pressure (mmHg)**
Optimal	<120	<80
Normal	<130	<85
High normal	130-139	85-89
**Hypertension**		
Grade 1 Hypertension (mild)	140-159	90-99
Grade 2 Hypertension (moderate)	160-179	100-109
Grade 3 Hypertension (severe)	≥180	≥110
**Isolated systolic/diastolic hypertension**		
Grade 1	140-159	≥90
Grade 2	≥160	≥100

Adopted from the British Hypertension Society guidelines.

Weight was measured using a pre-calibrated bathroom scale while wearing light clothing and barefoot. Weight was rounded off to the nearest 1 kg. Height was measured with the patient standing upright against a wall using a previously affixed height measuring device. Participants stood barefoot, with their backs, buttocks and heels in contact with the wall. Body Mass Index (BMI) of each patient was then calculated using the Table 2 and the formula:

$$\mathrm{BMI}=\frac{\mathrm{Weight}\;\left(\mathrm{kg}\right)}{\left[\mathrm{Height}\;\left(\mathrm{meters}\right)\right]2}$$

**Table 2:** International classification of adult underweight, overweight and obesity according to BMI

**Classification**	**Measurement (kg/m**^**2**^**)**
**Underweight**	**<18.50**
Severe thinness	<16.00
Moderate thinness	16.00 – 16.99
Mild thinness	17.00 – 18.49
**Normal**	**18.50 – 24.90**
**Overweight**	**≥25.00**
Pre-Obese	25.00 – 29.99
**Obese**	**≥30.00**
Class I	30.00 – 34.99
Class II	35.00 – 39.99
Class III	≥40

Adapted from WHO, 2000.

Obesity was defined as a BMI at least 30 kg/m^2^ based on the WHO guidelines of 2000.

Hip circumference was measured, by placing a dressmaker’s tape-measure horizontally at the point of maximum circumference over the buttocks, to the nearest 1 cm, with the respondent wearing light clothing.

Central obesity was defined as the proportion of waist circumference to hip circumference [waist-hip ratio (WHR)]. The waist circumference was measured in the mid-axillary line midway between the lower rib and the superior iliac crest using a standard stretch resistant dressmaker’s tape measure. Central obesity was defined as WHR above 0.85 and 0.95 for females and males respectively.

RBS was done on all patients using *SD CHeCK GOLD BLOOD GLUCOSE TEST MONITOR and STRIPS*. A patient with RBS of at least 11.1 mmol/L was classified as having diabetes and further management recommended. Patients with RBS between 7 mmol/L and 11 mmol/L were classified as a suspected diabetic and a fasting blood sugar test recommended [12].

Written informed consent was obtained from all patients while verbal consent was obtained from health workers. Permission was sought and granted from Provincial Medical Director (PMD), Mashonaland Central Province; District Medical Officer (DMO), Mazowe District; Health Studies Office (HSO), with ethical approval from the Joint Research and Ethics Committee (JREC/210/12) and Medical Research Council of Zimbabwe (MRCZ) approval number MRCZ/B/381.

A feasibility study was done at Tsungubvi clinic and relevant adjustments made to the final data collection tools.

Prevalence, frequencies, proportions, odds ratios, stratified Chi square tests and logistic regression analysis were done using Epi info 3.5.3 (CDC, Atlanta, 2011) and Microsoft Excel® 2007 was used to generate graphs. All calculations were made at 95% confidence interval (95% CI). Univariate analysis was initially done fitting information into previously prepared shell tables and graphs. This was followed by bivariate analysis to examine two variables comparing risk factors and outcomes. Stratified analysis was done for factors found to be statistically significant in the bivariate analysis to control for confounding and identify effect modification. *Stepwise logistic regression analysis* was then conducted to estimate measures of association of significant variables found in the bivariate analysis at the p = 0.25 level. We started with a single variable adding the other variables one by one at the 0.05 level (95% CI) eliminating all non-significant variables until all possible variables had been added.

## Results

### Introduction

**Figure 2:**
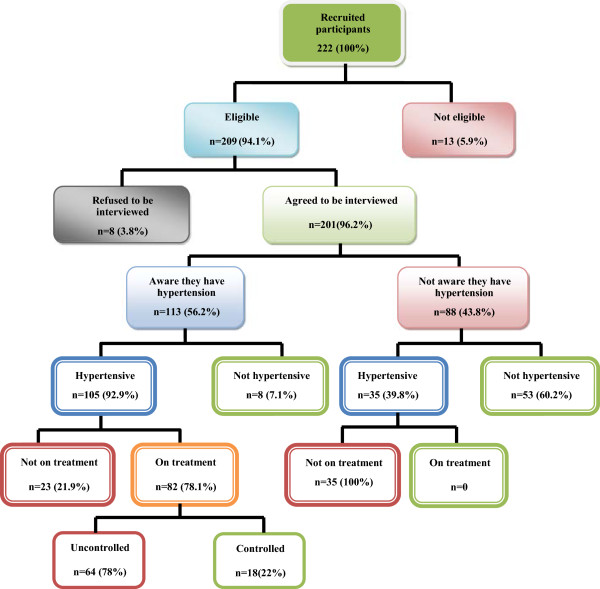
Flow chart of trends in the management of hypertension.

We interviewed 201 of 222 patients giving a response rate of 90.5%. Thirteen were excluded (non-residents of Mazowe) while 8 refused to participate (Figure 2). Socio-demographic details of patients are shown in Tables 3 and 4.

**Table 3:** Socio-demographic characteristics of patients

**Socio-demographic characteristics**	**Total (n = 201)**		**P-value**
	**No. hypertensive (140)**	**No. not hypertensive (61)**	
**Age group (years)**			
21-30	13	18	0.26
31-40	22	16	
41-50	40	11	
51-60	27	8	
>60	38	8	
**Sex**			
Female	94	36	0.27
Male	46	25	
**Marital Status**			
Married	103	47	0.08
Divorced	5	4	
Separated	10	1	
Widowed	20	5	
Single	2	4	
**Residence**			
Urban	42	18	0.17
Peri-urban	9	2	
Rural	62	20	
Farming	24	18	
Mining	3	3	
**Education Level**			
Never been to school	23	8	0.64
Primary level	64	24	
Secondary level	46	25	
Tertiary level	7	4	
**Occupation**			
Formal skilled	34	18	0.58
Informal	61	23	
Housewife	25	8	
Not employed	19	12	
Student	1	0	

**Table 4:** Socio-demographic characteristic factors affecting hypertension

**Socio-demographic characteristics**	**Total (n = 201)**		**POR (95% CI, lower – upper)**
	**Hypertensive (140)**	**Not Hypertensive (61)**	
	**(%)**	**(%)**	
**Age**			
>60 years	38 (84.4)	7 (15.6)	2.87 (1.2-6.87)*
≤60 years	102 (65.4)	54 (34.6)	
**Sex**			
Female	94 (72.3)	36 (27.7)	1.42 (0.76-2.64)
Male	46 (64.8)	25 (35.2)	
**Current marital status**			
Married	103 (68.7)	47 (31.3)	0.83 (0.41-1.68)
Not Married	37 (72.5)	14 (27.5)	
**Residence**			
Mainly urban	51 (71.8)	20 (28.2)	1.17 (0.62-2.22)
Mainly rural	89 (68.5)	41 (31.5)	
**Education level**			
At least secondary	53 (64.6)	29 (35.4)	0.67 (0.37-1.23)
Lower than secondary	87 (73.1)	32 (26.9)	
**Employment status**			
Employed	95 (69.9)	41 (30.1)	1.03 (0.54-1.96)
Not employed	45 (69.2)	20 (30.8)	

*Statistically significant.

### Prevalence and control of hypertension

The prevalence of hypertension was 69.7% (140/201) (95% CI: 62.8%-75.9%), based on SBP and or DBP. Hypertension was slightly more prevalent in women (72.3% vs. 64.8%: 95% CI: 0.76-2.63) though not significant (Table 4). Awareness was 75% (95% CI: 67–81.9%) with 82 (58.6%) of all hypertensive patients being on treatment. Of those on treatment 18 (22.0%: 95% CI: 12.7-31.5%) had controlled hypertension. Though not statistically significant, control was slightly better among women (22.6%) as compared to 17.9% in men (POR = 1.35; 95% CI: 0.42-4.30). The blood pressure values are shown in Table 5.

**Table 5:** Grading of systolic and diastolic BP of respondents in Mazowe District

**Classification of blood pressure**	**Diastolic blood pressure (%)**	**Systolic blood pressure (%)**
Optimal	32 (15.9%)	25 (12.4%)
Normal	26 (12.9%)	36 (17.9%)
High Normal	29 (14.4%)	32 (15.9%)
Grade 1 (mild)	53 (26.4%)	56 (27.9%)
Grade 2 (moderate)	43 (21.4%)	28 (13.9%)
Grade 3 (severe)	18 (9.0%)	24 (11.9%)
Mean values	92 ± 14.1 mmHg	145.7 ± 26 mmHg

### Rural vs urban

Most patients stayed in rural areas (40.8%) as compared to urban (29.9%) and farming (20.9%). The remainder were peri-urban (5.5%) and mining areas (3%). After reclassifying into mainly urban and mainly rural residence most patients were in the mainly rural category (64.7%; 95% CI: 57.6%-71.3%). The prevalence of hypertension was 71.8% (95% CI: 59.9%-81.9%) in urban areas and 68.5% (95% CI: 59.7%-76.3%) in rural areas. Though not significant, patients staying in urban areas were at higher risk of having hypertension as compared to those in rural areas (POR 1.17; 95% CI: 0.62-2.22). Awareness was comparable between patients staying in urban (72.5%) and rural (76.4%) areas (POR 0.82; 95% CI: 0.37-1.79) as well as being on treatment; urban (66.7%; 95% CI: 50.5%-80.4%) vs rural (74.6%; 95% CI: 62.9%-84.2%) with POR 0.68; 9% CI: 0.30-1.57. Control was better among patients from rural areas (25%) as compared to patients from urban areas (13.8%) but this was not statistically significant (POR 2.08; 95% CI: 0.61-7.1).

Awareness was higher among women in rural areas (79%) as compared to those in urban areas (71.9%) (POR 0.68; 95% CI: 0.25-1.81) while men in urban areas had higher awareness rate (73.7% vs 70.4% in rural men) (POR 1.18; 95% CI: 0.32-4.38). Treatment rate was higher among rural women (61.3%) and urban men (68.4%) as compared to urban women (50%) and rural men (55.6%). Control rate was higher among rural women (27%) and rural men (20%) as compared to urban women (12.5%) and urban men (15.4%).

### Characteristics of respondents

Patients’ median age was 45 years (IQR: 35 yrs - 60 yrs) with most being younger than 60 years (73.6%). Median ages were 48 years and 36 years for hypertensive and non-hypertensive patients respectively (*P* < 0.001). There were no statistically significant differences between hypertensive and non-hypertensive patients with respect to marital status (*P* = 0.082), place of residence (*P* = 0.174), highest level of education (*P* = 0.642) and employment status (0.585). Though not significant patients older than 60 years were: less likely to be aware of their hypertension (POR 0.64; 95% CI: 0.14-2.83), more likely to be on treatment for hypertension (POR 1.01; 95% CI: 0.41-2.52), less likely to have controlled hypertension among those on treatment (POR 0.47; 95% CI: 0.12-1.83).

### Knowledge of hypertension among patients

Most (70.6%) mentioned stress as a cause of hypertension. Clinical features mentioned were mainly headache and body weakness. Most (63.2%) thought stroke was the only complication of hypertension.

### Availability of health services

A high proportion of hypertensive patients were being attended to at local clinics (51.4%) as compared to hospitals (43.6%). Smaller proportions were consulting General Practitioners (3.6%) and traditional healers (1.4%) (*P* = 0.075).

Drugs such as nifedipine, captopril, methyldopa, propranolol, atenolol, prazosin, and digoxin were in available in small quantities or no available during the study period. Hydrochlorothiazide was available throughout the study period at all hospitals.

Howard Hospital had height measuring instruments fixed in both observation area and doctors’ consultation rooms though these were not being used. At Concession hospital the instrument was in pharmacy storeroom and not in use. All hospitals measured weight but not height.

Dress maker’s tape measures were available in all hospitals but not in use.

BP measurements were being done using aneroid sphygmomanometers in all hospitals. Glucometers were available but not used to routinely screen hypertensive patients for diabetes mellitus.

None of the health workers interviewed knew how to measure waist circumference, hip circumference or calculate WHR. There was no Information, Education and Communication (IEC) material or guidelines for management of hypertension for use in the health facilities. Clinicians were using the Essential Drugs List in Zimbabwe as a reference text.

### Overweight and obesity

The prevalence of overweight and obesity in the study population was 28.9% and 27.9% respectively. Central obesity was found in 50.8% of women and 7.0% of men. Forty-nine (35%) hypertensive patients were obese. Among hypertensive patients women were more likely to be obese than men (32.3% vs. 19.7%) with POR 2.55; 95CI: 1.13-5.75). Obesity was associated with hypertension (Chi-square 27.79; *P* < 0.001) with obese patients being 4.15 times more likely to be hypertensive than non-obese patients.

### Compliance and complications

Thirty-four (40.2%) hypertensive patients on treatment had ever missed at least two doses of medication. Patients missing doses were taking one 16 (48.5%); two 15 (45.5%); and three 2 (6.1%) types of antihypertensive >drugs. This did not affect compliance (Chi-square 0.85; *P* = 0.65). Reasons for missing doses were mainly drug side effects, trying alternative treatment, and feeling better.

Twenty-nine (20.7%, 95% CI: 14.3 – 28.4) hypertensive patients presented with a complication, such as congestive cardiac failure, visual problems, stroke or sensor y peripheral neuropathy. There were significant differences (20.7% vs. 3.3%) with hypertensive patients more likely to present with complications than non-hypertensive patients (POR 7.71; 95% CI: 1.78 – 33.4). Those with controlled hypertension were less likely to develop complications than those with poorly controlled hypertension (POR 0.31: *P* = 0.008).

### Co-morbidity

Eighty-three patients had co-morbid conditions. Ten percent had HIV while 12.1% (95% CI: 7.2-18.7) had diabetes mellitus. Eleven of the diabetic hypertensive patients were newly diagnosed. Diabetic patients were more likely to suffer from hypertension as compared to non-diabetic patients, however this was not statistically significant (POR 1.55; *P* = 0.41).

### Health service factors

Health workers measuring BP were not properly trained in all hospitals. Eight patients whose BP was said to be normal were found to have very high BP readings during the study. Common errors identified during BP measurements were patients extending their elbows whilst flexing their fingers, patients talking during BP checks, BP being checked with the cuff on top on clothing, patients holding the cuff to avoid it slipping off and only one reading being taken from the left arm.

Twenty-eight (20%) hypertensive respondents mentioned having been given advice on lifestyle modification. Only 36 (25.7%) and 10 (16.4%) hypertensive and non-hypertensive respondents had been screened for diabetes mellitus.

Twenty-four (11.9%) respondents were smokers. Of these only 4 were advised not to smoke. Hypertensive smokers were 15 (62.5%). Only 3 of these 15 were advised not to smoke. On the other hand 25 (14.1%) non-smokers currently hypertensive were advised not to smoke.

Consultation fees ranged from $0.00 to $10. Eighty-two respondents (58.6%) (40 Howard, 15 Concession and 27 Mvurwi) paid for consultation. Patients not paying fees had better hypertension control (POR 0.44; *P* = 0.007).

Twenty-three health workers, mostly Nurses and two Doctors, with a median 6 years in services were interviewed. Hypertension was correctly defined by 14 (60.9%) health workers. Only 4 (17.4%) correctly described the classification of hypertension. Knowledge on risk factors, symptoms and complications of hypertension was sought and the results are shown in Table 6. More than 50% of health workers knew obesity to be a risk factor for hypertension. Less than 50% of health care workers knew the other risk factors for hypertension as shown in Table 6. At least 13 health care workers knew that patients may present with headache, malaise or dizziness. In terms of complications more than 80% knew stroke to be a complication of hypertension.

**Table 6:** Assessment of health care worker knowledge on hypertension

**Category**	**Number of health workers giving response**	
	**Nurses**	**Doctors**
**Risk factors**		
Obesity	13	2
High salt intake	6	2
Increasing age	6	0
Alcohol abuse	3	0
Family history	2	1
Being female	1	0
**Symptoms**		
Headache	18	2
Malaise	15	0
Dizziness	11	2
Palpitations	7	0
Poor vision	9	2
Asymptomatic	0	2
**Complications**		
Stroke	19	2
Cardiac failure	8	2
Renal failure	4	2
Peripheral neuropathy	2	0
Blindness	2	0
Death	2	0

Various case scenarios were presented to health workers to assess how they were managing patients. The correct method of measuring BP was described by 10 (43.5%) health workers. A patient presenting with BPs of 130/85 and 150/90 were correctly managed by six (26.1%) and eight (34.8%) health care workers respectively. Sixteen health workers could correctly outline the monitoring of a patient on treatment for hypertension.

### Risk factors

As shown in Tables 4 and 3 risk factors associated with hypertension were age above 60 years (POR 2.87; 95% CI: 1.2-6.87), obesity (POR 4.15, 95% CI: 1.76-9.82), co-morbidity (POR 2.32; 95% CI: 1.23-4.39) and having high BP in the previous 12 months (POR 19.86; 95% CI: 8.61-45.9) (Table 7).

**Table 7:** Factors associated with hypertension

**Factor**	**Total (n = 201)**		**POR (95% CI, lower – upper)**
	**Hypertensive (140)**	**Not Hypertensive (61)**	
	**(%)**	**(%)**	
Knowledge of at least one cause			
Yes	105 (75)	44 (72.1)	1.16 (0.59-2.28)
No	35 (25)	17 (27.9)	
Family history of hypertension			
Yes	71 (50.7)	22 (36.1)	1.82 (0.98-3.39)
No	69 (49.3)	39 (63.9)	
Previous high BP			
Yes	105 (75)	8 (13.1)	19.86 (8.61-45.8)*
No	35 (25)	53 (86.9)	
Co-morbidity			
Yes	72 (51.8)	19 (31.7)	2.32 (1.23-4.39)*
No	67 (48.2)	41 (68.3)	
Obesity			
Yes	49 (35)	7 (11.5)	4.15 (1.76-9.82)*
No	91 (65)	54 (88.5)	

*Statistically significant.

Patients on treatment were more likely to have controlled hypertension than those who were not on treatment (POR 0.17; 95% CI: 0.09-0.31).

### Stratified analysis

Stratified analysis showed that the relationship between being on treatment and having controlled BP was *modified* by obesity as shown in Table 8. Obese patients were more likely to have poorly controlled BP.

**Table 8:** Association between being on treatment for hypertension and control of hypertension stratified by obesity

**Factor**		**Control of hypertension**		**Stratum specific POR (95% CI, lower-upper)**
		**Yes**	**No**	
Obese				
On treatment for hypertension	Yes	8	24	0.56 (0.18-1.76)
	No	9	15	
Non obese				
On treatment for hypertension	Yes	10	40	0.11 (0.05-0.25)
	No	66	29	
Crude				
On treatment for hypertension	Yes	18	64	0.17 (0.09-0.31)
	No	75	44	
Adjusted POR				
*P* < 0.001				

Sex also modified the effect of older age on development of hypertension with women being more likely to have hypertension than men (Table 9).

**Table 9:** Association between age above 60 years and risk for hypertension stratified by sex

**Factor**		**Hypertensive**	**Not hypertensive**	**Stratum specific POR (95% CI, lower-upper)**
Females				
>60 years	Yes	24 (25.5)	2 (5.6)	5.83 (1.30-26.11)*
	No	70 (74.5)	34 (94.4)	
Males				
>60 years	Yes	14 (30.4)	5 (20)	1.75 (0.55-5.61)
	No	32 (69.6)	20 (80)	
Crude				
>60 years	Yes	38 (27.1)	7 (11.5)	2.87 (1.20-6.87)*
	No	102 (72.9)	54 (88.5)	
Adjusted POR 3.07				
*P* = 0.011				

*Statistically significant.

### Multivariate analysis

Logistic regression analysis showed that independent determinants for developing hypertension in this study were age above 60 years, obesity and having a high BP reading in the previous 12 months as shown in Table 10.

**Table 10:** Factors associated with increased risk for hypertension among patients attending hospitals in Mazowe District: logistic regression analysis

***Risk factor***	***POR***	***95% CI***
Age group >60 years	**2.20**	**1.29-8.01**
Obesity	**3.30**	**1.25-9.0**
Having a previous high reading in last 12 months	**17.3**	**7.39-40.6**

## Discussion

Prevalence of hypertension was high in this study. Most patients were aware that they had hypertension and the majority were on treatment. There was, however, a gap between diagnosis and control of hypertension. The median age was higher among hypertensive patients as compared to the non-hypertensive patients.

Independent risk factors for hypertension found among patients were age above 60 years, obesity, high BP in the previous 12 months. Patients with hypertension were more likely to have complications associated with hypertension than non-hypertensive patients with the most common complication being cardiac failure.

Standard diagnostic tools such as dressmaker’s tape measure were either unavailable or not being used by health care workers. Simple errors in measuring BP were common among health care workers. Drug supply was erratic and dependent on the specific health centre with most patients relying on supplies from hospitals.

Prevalence of hypertension was expected to be higher than population based studies done in Zimbabwe and abroad but in this study it was much higher than expected. In addition to the rising prevalence of hypertension in Zimbabwe this may have also been due to the nature of the study, a hospital based study. Therefore the proportion of patients who present with hypertensive is likely to be higher than the population based studies in literature.

The prevalence of hypertension was slightly higher in women as compared to men, although this was not significant. We assume this may be due to the higher prevalence of obesity among women, a risk factor for hypertension. The STEPwise survey of 2005 also found a higher prevalence of hypertension in women. However there has been no consistence in the sex difference as shown by a study done in Mozambique where men had a higher prevalence of hypertension than women [7].

Prevalence of hypertension rose with increasing age. This is biologically plausible as the age related arteriosclerosis of blood vessels lowers flexibility of blood vessels causing the heart to increase the force of myocardial contraction raising BP. Similar to the study in Mozambique increasing age was associated with increased risk of hypertension [7].

Awareness of hypertension was very high in this study approaching proportions found in developed countries. The assumption is that study population had better health seeking behaviour. In addition all patients attending health facilities in Zimbabwe get BP checks where there are BP machines.

Control of hypertension was very low with no significant difference between men and women. Other studies have found better control of hypertension among women as compared to men [9,13,14]. With poor control of hypertension among patients the risk of complications is therefore likely to be higher. The poor control of BP in this study may have been worsened by the drug shortages experienced during the study period. A study by Morisky et al. showed that better control of BP was associated with a 57.3% lower risk of mortality among patients on treatment for hypertension through enhancing health educations among patients of treatment [15].

Though not significant in this study higher education levels have been noted to amount to better understanding of health related issues and better health outcomes. Due to societal norms women tend to be less educated than men. However, despite being less educated than men, women usually understand health messages better as they are in constant contact with the health delivery system more than men. According to Pandit et al. lower education attainment and more limited literacy are important predictors of poorer hypertension knowledge and control [16].

This realisation helps in development of educational tools to improve knowledge acquisition among patients.

There was a high proportion of undiagnosed hypertension in this study, probably due to the poor health worker practices found in all hospitals. This high proportion of patients with previously undiagnosed of hypertension is worrisome considering the fatal consequences of hypertension. In an American study 8% of the American hypertensives were undiagnosed [16]. In the PURE study it was noted that low rates of detection may be because few individuals have their blood pressure checked either through routine health assessment or screening programs and may be due to difficulties or costs in accessing health care [17]. According to the STEPwise survey up to 26% hypertensive people can develop a stroke without prior diagnosis of hypertension by health practitioners. Thus early diagnosis is vital.

The prevalence and awareness rate was higher in patients from urban areas while treatment and control rates were higher among patients from urban areas. This was however not significant. In comparison the awareness, treatment and control rates were significantly lower in patients from rural areas as compared to urban areas in the PURE study of which Zimbabwe was a part of.

In our study both SBP and DBP tended to be within the same grade in hypertensive patients older than 50 years. However, hypertensive patients younger than 50 years tended to have their DBP in a higher grade than SBP. Williams et al. showed that patients younger than 50 years tended to have either isolated DBP or both high systolic and diastolic BP in 30-40% of cases [18].

The Joint National Committee on Prevention, Detection, Evaluation and Treatment of High Blood Pressure has noted the importance of paying attention to SBP which is a major risk factor for cardiovascular diseases. According to the report most Physicians have been taught that diastolic pressure is more important than SBP thus they put more emphasis on DBP [19]. Simultaneously SBP has been largely ignored and considered part of the natural ageing process [20,21]. As a result health workers tend to concentrate more on the DBP alone. There is therefore need to re-focus attention of health workers in line with these findings.

Patient knowledge depends on the source of their information which is usually the local health practitioner. Stress has never been proven to be a direct cause of hypertension yet more than 70% of respondents believed it to be a cause. The only link between stress and hypertension is the temporary spikes in BP caused by stress via an upsurge of adrenaline from the adrenal glands [22,23]. This may, however, have an effect of the development of hypertension.

Health workers knowledge on complications of hypertension was poor therefore patients may present with complications which health workers may fail to identify. Zibaeenezhad also found that knowledge on hypertension was poor among health workers in his study where only 20% of Physicians could properly manage a patient with hypertension [24]. Another study in Pakistan found that junior and future Doctors had poor knowledge on hypertension [25]. Thus under such circumstances management of patients with hypertension is likely to be sub-optimal.

Increasing age and setting in of musculoskeletal problems like arthritis may make walking to health facility difficult for patients. There is therefore need to uphold the primary health care approach and avail drugs and services at clinic level, not just hospitals. Unfortunately quality of services is threatened by persistent resource shortage. The good working relationship between health workers and patients is a good starting point for improving patient knowledge and compliance to treatment and follow up.

Use of manual BP machine by poorly trained workers was one of the causes of poor diagnosis identified in this study. The WHO recommends use of digital BP machines to avoid such errors. Common avoidable errors were being made during BP measuring process in all hospitals resulting in missed diagnoses of hypertension. Simple tools for anthropometric measurements were not being used as health workers did not seem to understand the importance simple methods of assessing the cardiovascular risk of a patient. As a result patients may present late with complications like stroke, conditions that are very difficult to manage once they set in.

Advice on lifestyle modification has been given to most patients but hypertensive patients were more likely to benefit than non-hypertensive patients. This may mean health care workers are not promoting primary prevention of hypertension.

Consultation for chronic illnesses in Zimbabwe is free but patients in this study were made to pay. This may work against efforts to improve health care to hypertensive patients. Absence of drugs at clinic level worsens the situation for hypertensive patients as they are forced to travel to the hospitals. This study showed that free consultation enhanced control of hypertension making consultation fees an important barrier to care [26].

A study done in Ethiopia showed that co-morbidity affected compliance and adherence with those with more than one co-morbid conditions having poor adherence [27]. This was attributed to the pill burden. In Mazowe these factors were not significant predictors of poor compliance. Main reason for missing doses was drug side effects. With the poor health workers knowledge exhibited in this study it is highly unlikely that patients had adherence counselling prior to commencing treatment. This assumption may be supported by the fact that some respondents missed doses to seek alternative treatment as they believed their drug treatment was not effective or when they felt better.

Taking antihypertensive treatment has been linked to reduction of BP level and risk of stroke among patients [28]. In this study hypertensive patients on treatment were more likely to have lower BP than hypertension patients who were not on treatment. Unlike other studies, which have shown better control of hypertension, dual or triple therapy had no effect on control of hypertension in this study [27].

Obesity affects the effect of treatment on control of hypertension. This is because obesity increases insulin release which then thickens blood vessels in addition to release of renin and aldosterone. A study in USA showed that obese patients are at greater risk of hypertension and its complications when associated with a higher WHR. Literature has shown that obesity can result in a patient presenting with complications of hypertension without a previous diagnosis of hypertension. This may occur even at lower BP levels [22].

Risk factors found such as obesity are consistent with findings in other studies in Zimbabwe and other countries. In clinical trials obesity has been linked to cardiovascular risk factors such as diabetes mellitus and hyperlipidaemia. Hence weight reduction may work in the reverse direction to cause a decline in blood pressure irrespective of changes in salt intake as hypothesised by Hall et al. [22].

Complications were more prevalent than initially anticipated with cardiac failure being common. Visual defects due to hypertension may have been underestimated as there were no provisions for fundoscopy. Strokes were only 3.6% which is lower than initially anticipated.

A tenth of hypertensive patients had HIV. This is worrying as most Opportunistic Infections Clinic (OIC) staff tends to focus on HIV related ailments and ignore non-communicable diseases such as hypertension. This finding is very important as it enlightens the importance of measuring patients’ BP at every contact. In addition taking ARVs has been shown to induce metabolic syndrome where BP rises in association with abdominal adiposity.

Co-morbid diabetes mellitus was high. This is worrying considering 74.3% of hypertensive respondents had never been screened for diabetes prior to this study. Due to a hypothesised genetic link, most patients with poorly controlled hypertension are assumed to develop diabetes and vice versa [29,30]. It is therefore vital to screen and manage patients for diabetes mellitus.

Independent risk factors for hypertension are consistent with what is known about hypertension. Several studies have shown similar findings where increasing age and obesity were risk factors for developing hypertension. Central obesity was independently associated with poor control of hypertension for respondents on treatment. This is consistent with findings by Mahmood et al. in India [31,28].

Limitations included reluctance by local Doctors to participate in this study. During the study there was a tendency by other respondents to inform each other about the study. As a result there may have been an overestimation of the prevalence of hypertension in this study. Local Doctors tended to absent themselves on the days investigation was ongoing. Thus patients may have ended up participating in the study in an effort to get medical attention from the investigator. This may have distorted results of the study.

White coat hypertension could not be ruled out in this study.

We developed and used a non-standard system developed to grade health worker knowledge. The system assumed all responses were equally important which may not be the case in practice.

The drug supply and control system in the hospitals was not assessed in this study.

## Conclusions

The prevalence of hypertension in this hospital based study was very high with the burden being higher in those living in urban areas, women, the obese, and the elderly.

The diagnosis and management of hypertension is poor in Mazowe District with resultant high proportion of patients with undiagnosed hypertension. In addition a high proportion of patients on treatment are developing complications.

The absence of standard guidelines has made the situation worse. Contributing to these complications is the poor compliance to medications among hypertensive patients.

### Recommendations

We recommend that in the short term health workers need on the job training and mentorship in proper diagnosis and management of hypertension. Guidelines for the management of hypertension need to be printed and availed to health workers to help improve their knowledge, practices and to also serve as a reference.

Health workers need training in measuring and calculating WHR and BMI with guidance from the Non-Communicable Diseases Unit at MoHCC Head Office.

The Mazowe District Health Executive and Hospital Management Teams should prioritize purchase of digital BP machines.

In the medium term: National Pharmaceutical Company should provide drugs to health facilities. The Directorate of Pharmacy may need to relax the policy on second line antihypertensive drugs such as atenolol and avail them to clinics for resupply only to patients prescribed at higher levels.

The National Office must come up with appropriate awareness messages on hypertension and disseminate through the provincial and district offices.

The Province needs to set up Specialist NCDs clinics in the Province and ensure training of health staff.

In the long term: DHE should conduct operational research on hypertension and other non-communicable diseases in the district to enable evidence based practices. Available data on non-communicable diseases need to be analysed and used for decision making locally.

### Public health action

Subsequently the Directorate of Epidemiology and Disease Control drafted a policy document on non-communicable disease, a process leading to development of standard operating procedures for addressing the burden of non-communicable diseases in Zimbabwe. Currently the document is awaiting endorsement by parliament which will allow organizations such as the WHO to provide support for the directorate.

## Competing interests

The authors declare that they have no competing interests, both financial and non-financial.

## Authors’ contributions

MM: conception, design, acquisition, analysis and interpretation of data and drafting the manuscript. PM: conception, design, acquisition, analysis and interpretation of data and drafting the manuscript. LT: conception, design, data collection, analysis, interpretation and reviewing of several drafts of the manuscript for important intellectual content. NT: conception, design, reviewing of several drafts of the manuscript for important intellectual content. MT and SR had oversight of all stages of the research and critically reviewed the final draft for important intellectual content. All authors read and approved the final manuscript.

## Pre-publication history

The pre-publication history for this paper can be accessed here:

http://www.biomedcentral.com/1471-2261/14/102/prepub

## References

[B1] Cooper RS Rotimi CN Kaufman JS Muna WFT Mensah GA Hypertension treatment and control in sub-Saharan Africa: the epidemiological basis for policy BMJ 1998 316 614 617 9518920 10.1136/bmj.316.7131.614 PMC1112640

[B2] Gelfand M “Zimbabwe Africans’ Diet and Lifestyle”. Western Diseases: Their Emergence and Prevention 1981 Chicago: Edward Arnold Publishers Ltd 194

[B3] Kuller LH Epidemic hypertension in Sub-Saharan Africa Hypertension 2007 50 1004 1005 17954718 10.1161/HYPERTENSIONAHA.107.095620

[B4] Ganong WF Review of Medical Physiology 1993 16 San Francisco: Appleton & Lange

[B5] Akinkugbe OO Non Communicable Disease in Nigeria: Final Report of a National Survey 1997 Lagos: Federal Ministry of Health and Social Services

[B6] Williams B Poulter NR Brown MJ Davis M McInnes GT Potter JF Sever JF Thom SM British hypertension society guidelines for hypertension management 2004 (BHS-IV) BMJ 2004 328 634 640 15016698 10.1136/bmj.328.7440.634 PMC381142

[B7] Damasceno A Azevedo A Silva-Matos C Prista A Diogo D Lunet N Hypertension prevalence, awareness, treatment, and control in Mozambique: Urban/rural gap epidemiological transition Hypertension 2009 54 77 83 19470872 10.1161/HYPERTENSIONAHA.109.132423

[B8] Singh RB Sharma JP Rastogi V Niaz MA Singh NK Prevalence and determinants of hypertension in the Indian social class and heart survey J Hum Hypertens 1997 11 1 51 56 9111158 10.1038/sj.jhh.1000384

[B9] Hakim J Mujuru N Rusakaniko S Gomo ZAR National Survey, Zimbabwe non- Communicable Disease Risk Factors 2005 MoHCC, WHO, UNICEF 14

[B10] Mufunda J Scott LJ Chifamba J Matenga J Sparks B Cooper R Sparks H Correlates of blood pressure in an urban Zimbabwean population and comparison to other populations of African origin J Hum Hypertens 2000 14 1 65 73 10673734 10.1038/sj.jhh.1000886

[B11] Mufunda J Chatora R Nyarango P Chifamba J Sparks HV Prevalence of non-communicable diseases in Zimbabwe: results from analysis of data from the national central registry and urban survey Ethn Dis 2006 16 718 722 16937610

[B12] Patel P Macerollo A Diabetes mellitus: diagnosis and screening Am Fam Physician 2010 81 1 863 870 20353144

[B13] Pereira M Lunet N Azevedo A Barros H Differences in prevalence, awareness, treatment and control of hypertension between developing and developed countries J Hypertens 2009 27 5 963 975 19402221 10.1097/hjh.0b013e3283282f65

[B14] Kotchen JM Shakoor-Abdullah B Walker WE Chelius TH Hoffmann RG Kotchen TA Hypertension control and access to medical care in the Inner City Am J Public Health 1998 88 11 1696 1699 9807539 10.2105/ajph.88.11.1696 PMC1508561

[B15] Morisky DE Levine DM Green LW Shapiro S Russell RP Smith CR Five-year blood pressure control and mortality following health education for hypertensive patients Am J Public Health Feb 1983 73 2 153 162 10.2105/ajph.73.2.153 PMC1650522 6849473

[B16] Pandit AU Tang JW Bailey SC Davis TC Bocchini MV Persell SD Federman AD Wolf MS Education, literacy, and health: mediating effects on hypertension knowledge and control Patient Ed Counsell 2009 75 3 381 385 10.1016/j.pec.2009.04.006 19442477

[B17] Chow CK Teo KK Rangaraj S Islam S Gupta R Avezum A Bahonar A Chifamba J Dagenais G Diaz R Kazmi K Lanas F Wei L Lopez-Jaramillo P Fanghong L Ismail NH Puoane T Rosengren A Szuba A Temizhan A Wielgosz A Yusuf R Yusufali A McKee M Liu L Mony P Yusuf S Prevalence, awareness, treatment, and control of hypertension in rural and urban communities in high-, middle-, and low-income countries JAMA 2013 310 9 1 10 10.1001/jama.2013.184182 24002282

[B18] Williams B Lindholm LH Sever P Systolic pressure is all that matters? Lancet 2009 371 2219 2221 18561995 10.1016/S0140-6736(08)60804-1

[B19] US Dept of Health and Human Services The seventh report of the joint national committee on prevention, detection, evaluation and treatment of high blood pressure Nat High Blood Pressure Ed Progr 2004 http://www.nhlbi.nih.gov/files/docs/guidelines/jnc7full.pdf page 14-15, accessed 18/08/14

[B20] Sever PS Abandoning diastole BMJ 1999 318 1773 10381743 10.1136/bmj.318.7200.1773 PMC1116117

[B21] Williams B Lindholm LH Sever P Systolic pressure is all that matters Lancet 2008 371 2219 2221 18561995 10.1016/S0140-6736(08)60804-1

[B22] Hall JE da Silva AA Brandon E Stec DE Ying Z Jones DW Lip GYP, Hall JE Pathophysiology of Obesity Induced Hypertension and Target Organ Damage Pathogenesis: Genetics and Lifestyle 2007 New York: Elsevier 447 468

[B23] Zimmerman RS Frohlich ED Stress and hypertension J Hypertens Suppl 1990 8 4 103 107 2258776

[B24] Zibaeenezhad MJ Babaee H Vakili SH Knowledge, attitude and practice of general physicians in treatment and complications of hypertension in Fars province, southern Iran Iranian Red Crescent Med J 2007 9 1 4 8

[B25] Rehman A Shaikh MA Naqvi SAA Rehman T Awareness of hypertension among the medical students and junior doctors – a multicenter study from Pakistan J Pakistan Med Assoc 2011 61 1153 22126006

[B26] Moy E Bartman BA Weir MR Access to hypertensive care. Effects of income, insurance and source of care Arch Intern Med 1995 155 14 1492 1502 7605151

[B27] Dessie A Asres G Meseret S Birhanu Z Adherence to antihypertensive treatment and associated factors among patients on follow up at university of Gondar hospital, northwest Ethiopia BMC Public Health 2012 12 282 22490130 10.1186/1471-2458-12-282 PMC3395566

[B28] Tuck ML Sowers J Dornfield L Kledzik G Maxwell M The effect of weight reduction on blood pressure, plasma renin activity, and plasma aldosterone levels in obese patients N Engl J Med 1981 304 930 933 7010165 10.1056/NEJM198104163041602

[B29] Ong KL Wong LY Man YB Leung Y Song Y Lam KSL Cheung BMY Haplotypes in the urotensin II gene and urotensin II receptor gene are associated with insulin resistance and impaired glucose tolerance Peptides 2006 27 1659 1667 16597476 10.1016/j.peptides.2006.02.008

[B30] Chow WS Cheung BM Tso AW Xu A Wat NMS Fong CHY Tam Y Tan KCB Janus ED Lam TH Lam KSL Hypoadiponectinemia as a predictor for the development of hypertension: a 5-year prospective study Hypertension 2007 49 1455 1461 17452504 10.1161/HYPERTENSIONAHA.107.086835

[B31] Mahmood SE Srivastava A Shrotriya VP Shaifali VP Mishra P Prevalence and epidemiological correlates of hypertension among labour population Nat J Community Med 2011 2 1 43 48

